# On Calibrating the Sensor Errors of a PDR-Based Indoor Localization System

**DOI:** 10.3390/s130404781

**Published:** 2013-04-10

**Authors:** Kun-Chan Lan, Wen-Yuah Shih

**Affiliations:** 1 Department of CSIE, National Cheng Kung University, Tainan 701, Taiwan; 2 Department of CS, National Chiao Tung University, Hsinchu 30010, Taiwan; E-Mail: todd629.cs01g@nctu.edu.tw

**Keywords:** pedestrian dead reckoning, waist-mounted, simple harmonic motion, ZUPT, map matching, floor plan

## Abstract

Many studies utilize the signal strength of short-range radio systems (such as WiFi, ultrasound and infrared) to build a radio map for indoor localization, by deploying a large number of beacon nodes within a building. The drawback of such an infrastructure-based approach is that the deployment and calibration of the system are costly and labor-intensive. Some prior studies proposed the use of Pedestrian Dead Reckoning (PDR) for indoor localization, which does not require the deployment of beacon nodes. In a PDR system, a small number of sensors are put on the pedestrian. These sensors (such as a G-sensor and gyroscope) are used to estimate the distance and direction that a user travels. The effectiveness of a PDR system lies in its success in accurately estimating the user's moving distance and direction. In this work, we propose a novel waist-mounted based PDR that can measure the user's step lengths with a high accuracy. We utilize vertical acceleration of the body to calculate the user's change in height during walking. Based on the Pythagorean Theorem, we can then estimate each step length using this data. Furthermore, we design a map matching algorithm to calibrate the direction errors from the gyro using building floor plans. The results of our experiment show that we can achieve about 98.26% accuracy in estimating the user's walking distance, with an overall location error of about 0.48 m.

## 1. Introduction

The accurate localization of objects and people in an environment has long been considered an important component of ubiquitous networking. There are many studies on indoor localization [1,2], several of which involve the use of radio signal strength indicators (RSSI) [3–8] and a large number of preinstalled devices, called beacon nodes, which periodically emit signals. By listening and monitoring signal changes [9,10], the user's location can be estimated. While the idea of these existing systems is attractive, they require intensive pre-deployment efforts for every new building. In addition, the signal variations over time could be potentially very large for real-world operations [11].

On the other hand, a personal dead reckoning or pedestrian dead reckoning (PDR) system is a self-contained technique for indoor localization. This technique only requires a couple of sensors to be put on the user, so that it can be used in any building without pre-installing beacon nodes or pre-building RF maps/propagation models based on surveys of the environment. Most PDR systems use inertial sensors (such as an accelerometer, gyroscope, or digital compass) to measure step length and heading direction. Some also use other sensors, such as cameras [12], ultrasound [13], RFID [14,15], or laser [16], to calibrate their results. Generally, depending on where the sensors are placed, previous studies classified PDR systems into two types: foot- and waist-mounted. The foot-mounted method [17–27] uses a double integral on horizontal acceleration to estimate distance, and a gyroscope or compass to measure the heading direction. On the other hand, the waist-mounted [28–31] method tries to detect each step event to calculate the total number of steps, and then multiplies this by a constant step length which is based on the pedestrian's characteristics (weight, height, and age) to estimate the total moving distance. Some waist-mounted methods use linear regression to find the relationship between the acceleration, walking speed, and step length [32]. However, when the pedestrian's walking pattern is different from the predefined step length model, this may adversely affect the distance estimation. Finally, although a waist-mounted method is generally more feasible to be implemented on a hand-held device, its accuracy in estimating the step length is typically worse than that of a foot-mounted method which, on the other hand, performs poorly with regard to obtaining an accurate orientation [33].

**Figure 1.**
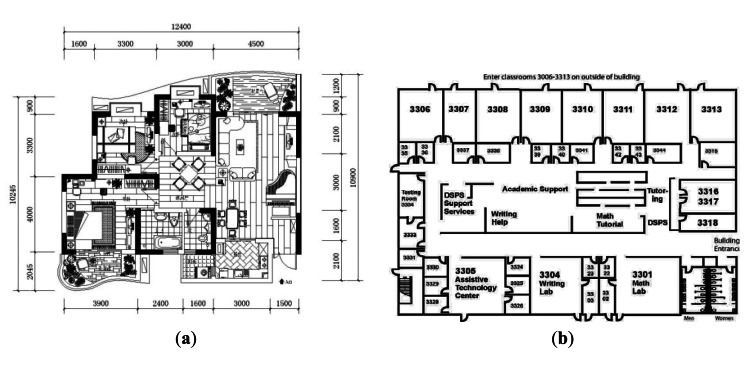
(**a**) A detailed map and (**b**) a floor plan.

Sensor drift [34] is a well-known problem in PDR systems. Given that the hardware used in such systems is not perfect, the inertial sensors constantly have some small errors when estimating the distance and direction, and signal noise (such as vibrations of the user's body) can further exacerbate this problem [25,26]. Some PDR systems use a map matching mechanism to calibrate these errors [35–37], and these can be categorized into two types. One tries to match the user trajectory to the closest junction and road on the map [37], while the other utilizes the map to filter out positions where the user is unlikely to move (e.g., walls, obstacles, and so on) [35,36]. Both techniques require the use of a detailed scaled map of the building, as shown in Figure 1(a), although in practice this is usually difficult to obtain.

**Figure 2.**
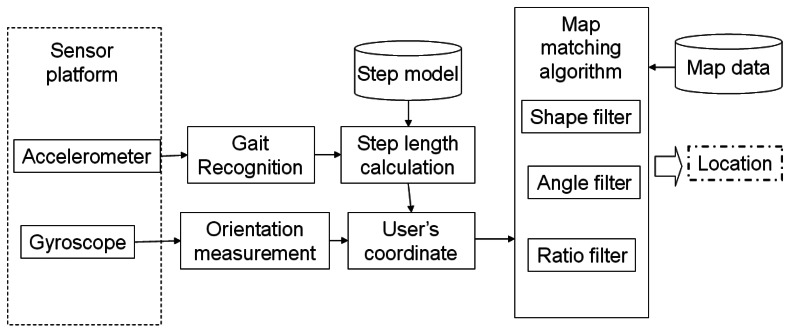
The entire system architecture.

In this paper, we consider a scenario in which the user has a smart-phone and can access the floor plan of the building, like the one shown in Figure 1(b), as these are widely available. The system utilizes the sensors on the smart-phone to compute the user's moving distance and direction, and this can be used with the map to estimate their current position in the building, as shown in Figure 2. Our system can be considered as an add-on to a traditional outdoor navigation app (e.g., Google Latitude [38]) so that the starting position of the user in the building can be estimated using the last-recorded GPS position.

The contribution of this paper is threefold. First, using the characteristic of simple harmonic motion (SHM) and the Pythagorean Theorem, we propose a novel waist-mounted PDR method to accurately estimate the step length for measuring the moving distance. The accuracy of our distance estimation is about 98.25%. Second, based on the geometric similarity between the user trajectory and the floor map, we design a map-matching algorithm to calibrate sensor errors using common building floor plans. Finally, we implement our PDR system on a smart-phone, and our results show that the location error is only about 0.48 m in our test scenario.

The rest of this paper is structured as follows: In Section 2, we describe the related work. We discuss the details of our step-length estimation and map-matching algorithms in Section 3 and Section 4, respectively. The results of our experiment are shown in Section 5. Finally, we conclude this paper in Section 6.

## 2. Related Work

Our work is built on some previous studies of indoor localization, step length estimation, and map matching.

### 2.1. Indoor Localization

Many studies of indoor localization use wireless signals to estimate the location, and such approaches can generally be divided into three types, those that use triangulation, fingerprinting, and proximity. The triangulation methods include, for example, the use of time of arrival (TOA) [39], time difference of arrival (TDOA) [40], and received signal strength (RSS) [3–9]. Fingerprinting methods also utilize the RSS, and in these, a survey phase is required to measure and record the RSS of every place in the building to build a radio map. To locate the target's position, one simply measures the RSS at the user's location and uses some pattern-matching algorithms, such as KNN [41] or SVM [42], to find the position in the radio map which has the most similar RSS record. Finally, the proximity methods usually first deploy many antennas (e.g., RFID [43–45]) at known positions. The antenna which is closest to the mobile target will generally have the strongest RSS, which can then be used to estimate the location of the target. However, these signal-based approaches suffer a common problem, which is that radio signals are very often suffer from interference in an indoor environment [11]. In addition, the deployment of the necessary infrastructure (e.g., base-stations or antennas) is costly and labor-intensive.

In addition to the signal-based methods, many prior studies use a PDR system to estimate the trajectory of a user by placing some sensors on the user's body. Inertial sensors, such as accelerometers, gyroscopes, and compasses, are commonly used in such systems. Some PDR systems also include GPS sensors [46,47], and use these to calibrate the PDR drift as long as the GPS signal is available. When the GPS signal is obstructed, the system can then be changed to the PDR mode and continue to record the trajectory. Our study is based on the PDR system, which has the advantage of avoiding the deployment overhead of the signal-based methods. On the other hand, the performance of our system can be enhanced by a signal-based system if available. For example, one limitation of our map-matching approach is its need to collect “enough” trajectory data before it can uniquely identify the user's position on the map. When there is not enough trajectory information, we make use of the known locations of existing WiFi base stations in the building to help estimate the user's location [48–50].

### 2.2. Step Length Estimation

A PDR system can be classified into two types depending on where the sensor is mounted: foot-mounted [17–27] and waist-mounted [28–31]. During walking, when one foot is swinging forward, the other one must stand on the ground to support the weight of the body, and both methods [17–27] use these movement characteristics to detect a step event. To calculate the step length, the foot-mounted methods typically perform a double integral on the horizontal acceleration. However, without further calibration, the problem of sensor drift [34] could introduce serious inaccuracies when estimating the step length. One way to calibrate the sensor drift error is called zero velocity update (ZUPT). When the swinging-foot touches the ground, the angular velocity of this foot will be close to zero, which can be used to reset the system to avoid sensor drift errors accumulating into the length estimation of the next step.

On the other hand, for a waist-mounted PDR system, ZUPT is not directly applicable, since one will not be able to find zero velocity in the horizontal direction. Some waist-mounted PDR systems use a constant step length [51–54] while the others [30,55] use a trace-driven approach, by first collecting empirical data from multiple users and then using linear regression to find the relation between step length, walking frequency, and the variance of acceleration. The limitation of this approach is that one might need to collect new training data for a new user. Weinberg [31] observed that the upper body moves vertically when walking, and suggested that one can estimate the step length as follows Equation (1):

(1) StepLength=2×heightchan ge/α

where *α* is the swinging angle of the leg from the body, and the *heightchange* can be estimated based on the vertical acceleration. However, he did not discuss how to measure *α*. Our idea is similar to Weinberg's, but we estimate the step length based on the height change and length of the leg using the Pythagorean Theorem. In addition, we use the concept of Simple Harmonic Motion (SHM) to find the zero velocity in the vertical direction and apply ZUPT to avoid the accumulation of sensor drift errors on the vertical-axis.

### 2.3. Map-Matching Algorithm

In some prior PDR systems, a map-matching mechanism is used to match the user trajectory onto the map [35–37] in order to calibrate the sensor errors. There are two ways this is achieved. The first tries to match the user trajectory to the closest junction and road on the map [37], while the other utilizes the map information to filter out positions where the user is unlikely to walk (e.g., walls and obstacles) [35,36]. However, both techniques require the use of a detailed scaled map of the building (*i.e.*, with detailed distance information for each route on the map) which, however, is usually not easy to obtain in reality. In our work, we utilize the more commonly-seen building floor plans instead of detailed scaled maps. Based on the geometric similarity between the trajectory data and the map, we propose a new map-matching method that uses the floor plan for locating the user. As shown later in Section 5, our approach has similar performance to those methods that rely on detailed scaled map information.

## 3. A Waist-mounted Pedestrian Dead Reckoning (PDR) System

**Figure 3.**
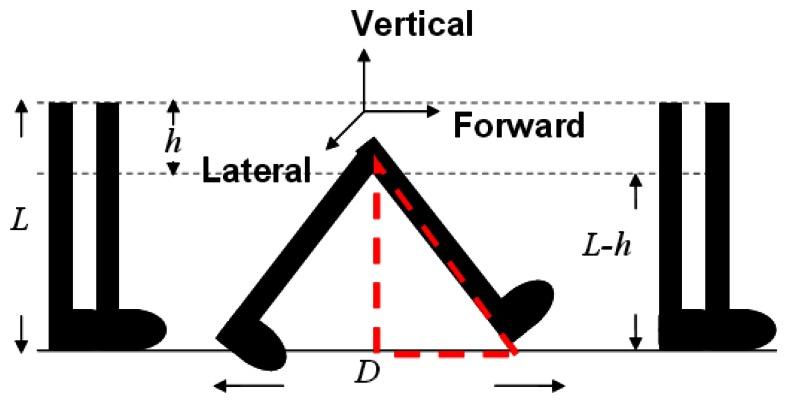
Walking Diagram.

In this section, we describe the design of our waist-mounted method for estimating the user's walking step length. We observe that, as shown in Figure 3, when the sensor is mounted on the waist, the height of the sensor (*i.e.*, *L-h*, where *h* is the vertical displacement of the sensor during the walking), the length of the leg (*i.e.*, *L*) and half of the step length (*i.e.*, *D*/*2*) forms a right triangle in which leg length is the hypotenuse. Assuming that *L* is known, based on the Pythagorean Theorem, we can obtain step length *D* if we can obtain the vertical displacement of the sensor (*i.e.*, *h*), and details of this are discussed later in this section.

**Figure 4.**
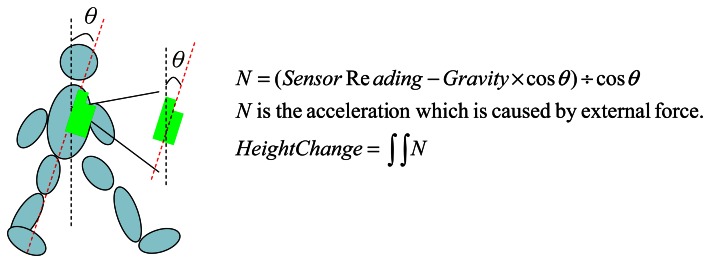
The orientation of the device.

**Figure 5.**
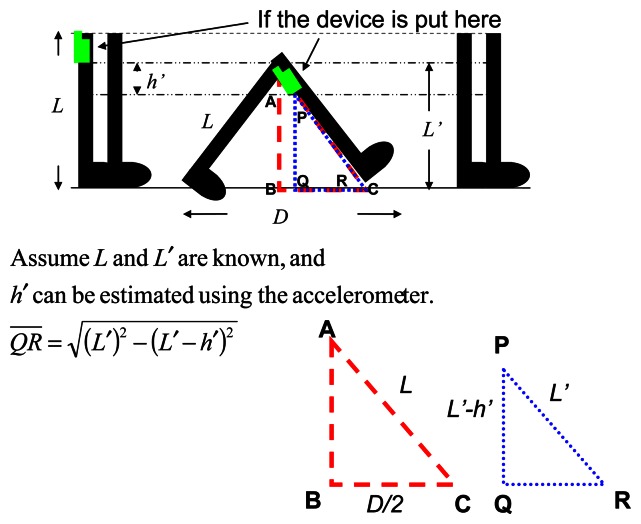
The diagram of device in the lower body.

We focus on the waist-mounted method because it is generally more feasible to be extended for a hand-held device, such as a smart-phone. When implementing a PDR system on a hand-held device, two cases can be considered. The first is when the user holds the device (e.g., talking on the phone), and the other is when the device is put in a pocket or a bag. Prior research [56] has shown the feasibility of using a waist-mounted method for the first case. Therefore, in this section we only discuss how to extend the results of a waist-mounted method for the second case. To implement a waist-mounted method on a hand-held device, two issues need to be considered: orientation and placement of the device. The orientation of the device may change from time to time when it is put in a pocket or bag during walking, and this change in orientation affects the influence of gravity on the three axes of the accelerometer, and results in different readings from the sensor. To resolve this problem, we can adopt a method similar to that in an earlier work, Su *et al.* [56] which used a gyroscope to record changes in orientation and to calibrate the system, as shown in Figure 4. Next, in a waist-mounted method, the vertical movement displacement of the device is the key parameter to estimate the step length. When the device is positioned above the waist-line, its vertical displacement during walking will be the same as when it is mounted on the waist. On the other hand, when the device is placed below the waist-line (*e.g.*, in a pants pocket), we can estimate the vertical displacement of the waist as follows. Assuming the leg length (*L*) and the pocket position from the ground (*L′*) are already known, we can find two similar triangles *ΔABC* and *ΔPQR*, as shown in Figure 5. Based on the Pythagorean Theorem, we can obtain $\bar{QR}$ by first measuring *L′* and *h′* (*i.e.*, vertical displacement of the pocket during the walking). Then, using triangle similarity, we can find $\bar{BC}=D/2=\left(L\times \bar{QR}\right)/L\prime \cdot h\prime$ can be measured in a similar way as to measure *h*, which is discussed next.

### 3.1. Using the Height Change of the Waist to Estimate Step Length

Previously Weinberg [31] proposed that one can estimate the step length using the height change of the waist and the *swinging angle of the leg* during walking [31]. However, he did not discuss how to measure this angle, and we found that, in practice, it is difficult to measure such a small angle during walking. In this paper we use the Pythagorean Theorem and propose a different way to estimate step length using the change in height. During walking, a person's body moves up and down. If we assume the length of the leg is *L*, the waist-line will move up-and-down between *L* and *(L-h)* from the ground, where *h* is the change in the height of the waist. Consider the triangle in Figure 3, formed by the two feet of a person and their step length *D*. Given that *L* is known, by using the Pythagorean Theorem, we can estimate *D* if we know the height of this triangle, *i.e.*, *(L-h)*. To obtain *(L-h)*, we need to first calculate *h* which is the change in height of the waist during walking. Therefore, if we mount an accelerometer on the user's waist, the readings of the accelerometer can be used to estimate the height change *h*, which can then be used to calculate the step length *D* based on the Pythagorean Theorem.

To implement this method, we need to first resolve the following issues.

(1) How to eliminate the influence of gravity?
(2) How to remove signal noise, such as vibrations from the body?
(3) How to recognize the beginning and end of a step based on readings from the sensor?
(4) How to prevent sensor drift errors accumulating from one step to the next?

**Figure 6.**
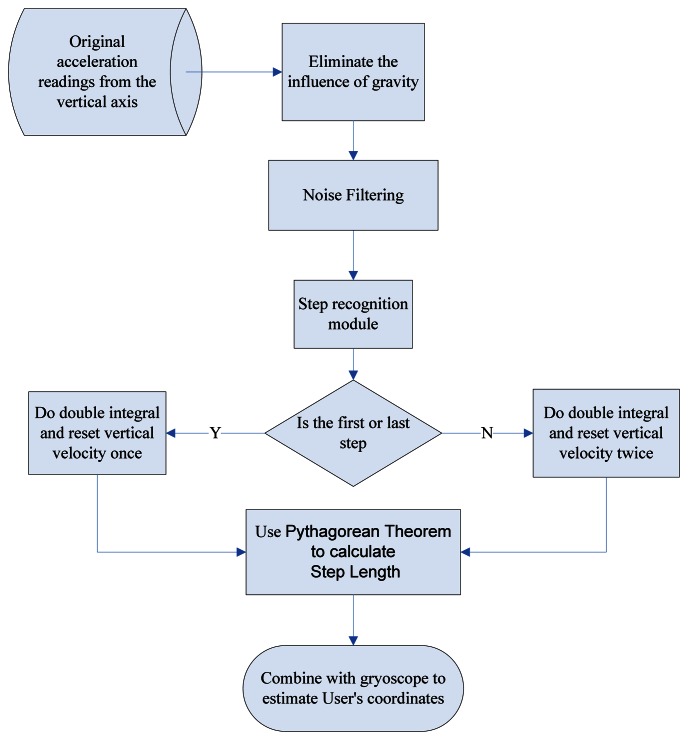
Flow Chart of PDR.

Our system architecture is shown in Figure 6. We first remove the influence of gravity from the original sensor data, and then the noise is filtered out by a low-pass filter. The filtered signal is fed into the step recognition module to identify each new step. We adopt the concept of simple harmonic motion (SHM) [57] to reset the vertical velocity at the beginning of each new step, which prevents sensor drift errors from being accumulated over to the next one. Finally, the step length is estimated based on the filtered sensor data and the foot length.

### 3.2. The elimination of the Influence of Gravity

The readings from an accelerometer are affected by gravity. An accelerometer is typically built on a silicon wafer, with a poly-silicon spring on the wafer surface to provide an opposing force against external force. Any external force, including gravity, can cause the deflection of the spring. According to the different levels and direction of this deflection, the values of resistance or capacitance on the circuit will change proportionally, and the device will then output the corresponding voltages, which are the readings measured from the sensor. Therefore, at any time, the sensor can detect the acceleration due to gravity (1 g on Earth), except when the axis (we use a three-axis accelerometer) from which we collect the data is perpendicular to gravity. Therefore, to obtain the vertical acceleration of the waist during walking for estimating changes in height, we first need to remove the effect of gravity from the sensor readings. Generally speaking, the angle between the direction of the measured axis and the direction of gravity can be from 0° to 180°. For the sake of discussion, we consider two cases: when the axis is parallel to the direction of gravity and when it is not.

#### 3.2.1. If the Axis and the Direction of Gravity Are Parallel

**Figure 7.**
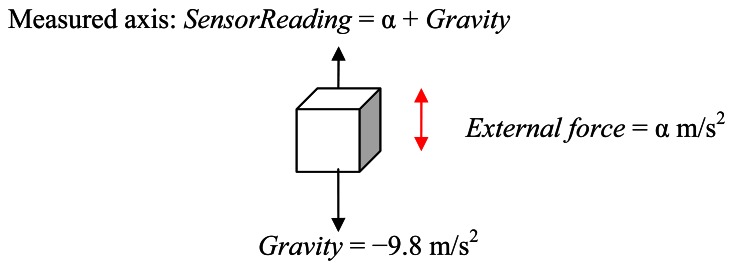
Gravity Elimination.

In this case, the angle between the measured axis and the direction of gravity is 0° or 180°, as shown in Figure 7. The vertical acceleration caused by the up-and-down movement of the waist during walking can be estimated by Equation (2). Here, *SensorReading* is the reading we collect from the sensor for one particular axis, while *α* is the external force that causes the sensor to move up or down. The minus sign indicates that the direction of the force is downward.

(2) $$\alpha =\text{Sensor Reading}-\text{Gravity}(=-9.8m/s^{2})$$

Note that since gravity is a downward force, the reading *α* from the sensor will be more than 1 g (−9.8 m/s^2^).

#### 3.2.2. If the Axis and the Direction of Gravity Are Not Parallel

**Figure 8.**
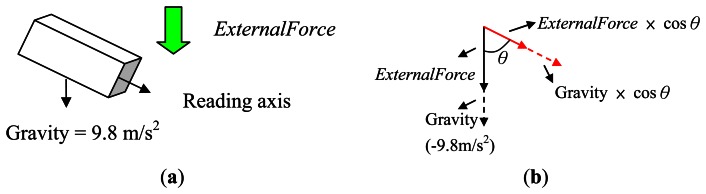
(**a**) Gravity elimination in different directions; (**b**) Force Diagram.

As shown in Figure 8(a), the measurements collected from the sensor will be affected by two different forces: gravity and the external force, as shown in Figure 8(b). Here we assume that the external force comes from the up-and-down movement of the waist during walking. To estimate the external force, we need to first compute the component of the external force on the measured axis, which can be achieved by subtracting the component of gravity from the sensor reading. Assuming the angle between the direction of measured axis and the direction of the gravity is *θ*, we can then obtain Equation (3.3):

(3.1) M=gravity×cosθ

(3.2) N=ExternalFo rce

(3.3) Sensor Reading=N×cosθ+M

In the above equation, *SensorReading* is the value read from the measured axis of the sensor, which is the sum of the component of *ExternalForce* and the component of gravity. *M* is the gravity component which can be measured when the sensor is static (e.g., placed on a table):

(3.4) N=(Sensor Reading−M)÷cosθ

(3.5) $$\theta =\cos^{-1}(M/\text{gravity})$$

The external force *N* can then be calculated using Equations (3.4) and (3.5).

(3.6) $$N=(\text{SensorReading}-M)\times \sec(\cos^{-1}(M/9.8))$$

### 3.3. Noise Filtering

During walking, some unexpected and unpredictable body vibrations might cause some higher-frequency noise in the sensor readings. In this section, we discuss how we filter such noise.

Intuitively, one can use a low pass filter and preset a cut-off frequency to filter the noise. Some prior work has shown that the frequency of human muscle movement is lower than 16 Hz [58], and the human step frequency is never higher than 3 Hz [29]. Therefore, some step recognition systems use 3 Hz as their cut-off frequency to filter noise signal [53]. Initially, we also used 3 Hz, but then found our results became worse. This shows that, while it is good enough to detect a new step event, a 3 Hz threshold is too low for our purposes, and will remove data which is not noise. Some prior work [59] analyzed the acceleration of the waist during walking, and found the maximum acceleration is 8 Hz. Therefore, we set our cut-off frequency at 8Hz for filtering the noise, and this threshold worked well under repeated experiments with different subjects.

### 3.4. Step Recognition Module

**Figure 9.**
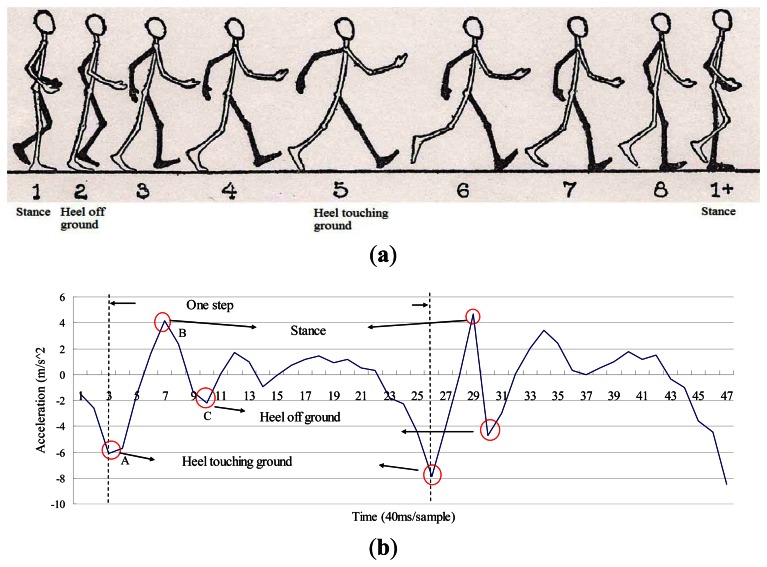
(a) Walking diagram (modified from an earlier work [60]); (b) The vertical acceleration of walking.

In order to recognize a step, we first analyze the components of one step that can cause vertical changes to the body. There are three major events which may affect the height of the waist, as shown in Figure 9(a).

The first one is a heel-touching-ground event, which happens when the heel just hits the ground and the waist is in its lowest position during the entire step. The event that comes after this is the stance, which occurs when the foot is flat on the ground. Finally, the heel-off-ground event occurs right after the stance. Generally, as shown in Figure 9(b), the vertical acceleration of a heel-touching-ground event is the local minimum within a step. In addition, previous studies [51,61] showed that human walking frequency is never over 3 Hz. Therefore, the duration between two consecutive heel-touching-ground events must be over 0.33 second. Based on the above, we use a sliding window algorithm to detect every heel-touching-ground event, and define one step as from a heel-touching-ground event to the next heel-touching-ground event. Once a new step is identified, the sensor data between two consecutive heel-touching-ground events will be used to estimate the step length.

### 3.5. Step Length Estimator

#### 3.5.1. Our Step Model

To measure the walking distance from point A to point B, we sum up the step length of all steps. We model a complete step as from one heel-touching-ground-event to the next. Therefore, we consider the first step [*i.e.*, from the stance event to the heel-touching-ground event, as shown in Figure 9(a) ] and the last step (*i.e.*, from the heel-touching-ground event to the stance event) as half a step. When the first or last step is detected, our system will divide the calculated step length by 2.

#### 3.5.2. Simple Harmonic Motion

Once each step can be identified, we can then do the double integral to calculate the height change of the waist and then use this information to estimate the length of each stride based on the Pythagorean Theorem. However, as discussed previously, if the system only naively does the double integral on the accelerometer data, the sensor drifts errors could accumulate from one step to the next. To avoid this, we propose a zero velocity update method to calibrate the sensor data based on the concept of Simple Harmonic Motion (SHM) [57].

Simple harmonic motion has been widely used to model various physical phenomena, such as the oscillations of a spring. When an object is in simple harmonic motion, the displacement of the object is proportional to the external force placed on it, and the force always points to the position of equilibrium. In other words, when the object is displaced from its equilibrium position, it experiences a net restoring force toward this position. One characteristic of SHM is that when an object is at its largest displacement from the equilibrium position, the object's speed will become zero, and at the same time, the object will reach its maximum acceleration rate. For instance, if we put a pen on a spring and make the spring oscillate in vertical way, and then put a long ribbon of paper on a table and let the pen draw on it to show the trajectory of spring vibration at the same time as we pull the paper in a horizontal direction at a stable speed, then the trajectory shown on the paper will look like a sinusoidal wave, and the vertical velocity of the highest and the lowest points of the spring will be zero.

Inspired by this observation, if we mount a sensor on the waist of a pedestrian, then while they are walking the trajectory of the sensor can be approximated by a sinusoidal wave. In addition, given that the velocity of the highest and lowest points of the sensor will be zero, we can utilize these characteristics to detect when the user starts a new step. Finally, once the points with zero velocity in the vertical direction [where the vertical acceleration reaches its local maximum, *i.e.*, points A and B in Figure 9(b) ] are identified, we can then reset the vertical velocity to zero before doing the double integral for calculating the height change of the user's waist. The height change can then be used to estimate the step distance based on the Pythagorean Theorem.

#### 3.5.3. Step Length Estimation

Once a new step event is detected, we can compute the step length using the double integral: the integral of acceleration will give us the velocity and we can get the distance of the step from the integral of the velocity. To avoid sensor drift errors being accumulated, which would adversely affect the double integral results, we can utilize the abovementioned characteristics of SHM to calibrate the sensor data. As discussed previously, there are three major events during one step, namely heel-touching-ground, stance, and heel-off-ground. As shown in Figure 9(b), the lowest valley (point A) of the wave indicates when the heel is touching ground [corresponding to the 5th posture in Figure 9(a) ], the first peak occurs (point B) when the walker is in the stance state [corresponding to the first posture in Figure 9(a) ]. Following the stance event, the body starts to lean forward and the foot is now on its toes, which will give a force to push the body up, so that the vertical acceleration will change to the opposite direction and cause the second valley in Figure 9(b) [*i.e.*, point C, corresponding to the second posture in Figure 9(b)], due to the law of inertia.

**Figure 10.**
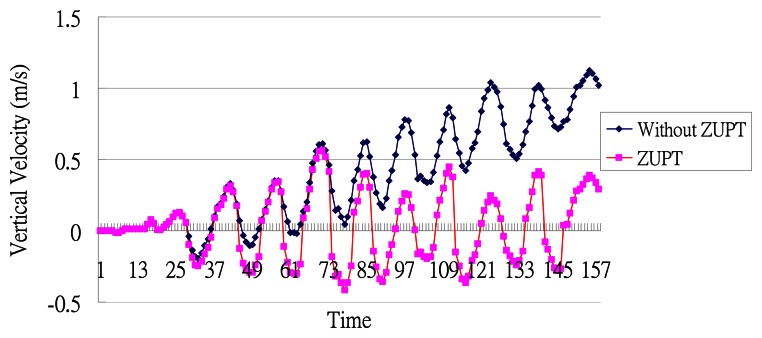
The velocity with ZUPT versus without ZUPT.

As shown in Figure 9(a), when the stance and heel-touching-ground events occur, the waist has the largest displacement from its equilibrium position. Therefore, we reset the vertical velocity to zero at these points. We performed an experiment to observe the effect of doing such a zero velocity update (ZUPT). As shown in Figure 10, implementing ZUPT can indeed avoid the accumulation of sensor drift errors:

(4) $$V_{n}=\int_{1}^{n}a_{n}\;dt$$

(5) $$h=(\int_{1}^{n}\left|V_{n}\right|dt)/2$$

Next, since we consider the change in height of the waist as a simple harmonic motion, if we simply do the double integral without considering the direction of the force (*i.e.*, the height change is due to the ascent and descent of the body movement), the computed displacement will be zero. Therefore, we calculate the absolute value of current velocity and use it to do the next integral, as shown in Equations (4) and (5), where *V_n_* is the vertical velocity, *a_n_* is the vertical acceleration, and *h* is the change in height. We can get the velocity by doing the integral of vertical acceleration. The result of this is then divided by two to get an average of height change; here we assume that the height change in the directions of ascent and descent are approximately the same. Finally, we use the obtained height change of the waist to estimate stride length based on the Pythagorean Theorem. As shown in Figure 3, if we assume that the leg length is *L* and the height change is *h*, we can use Equation (6) to calculate the step distance *D*:

(6) $$D=2\times \sqrt{L^{2}-{\left(L-h\right)}^{2}}$$

The first and last steps are considered as special cases. If the system detects that the user is currently in his/her first or last step, the height change will not be divided by two. In addition, there is only one point where the velocity needs to be reset, because the initial velocity of the first step is already zero and the last step does not need to reset this when the body is in its stance state. Combining the distance and the orientation information (which can be obtained from a gyroscope), we can then calculate the user's coordinates and trajectory in a 2D space, as shown in Equation (7):

(7) $$\left\{ \begin{array}{l} X_{n}=X_{n-1}+Dis\times Cos\theta \\ Y_{n}=Y_{\;\;n-1}+Dis\times Sin\theta \end{array}\right.$$

#### 3.5.4. Moving at High Speed or on the Same Spot

**Figure 11.**
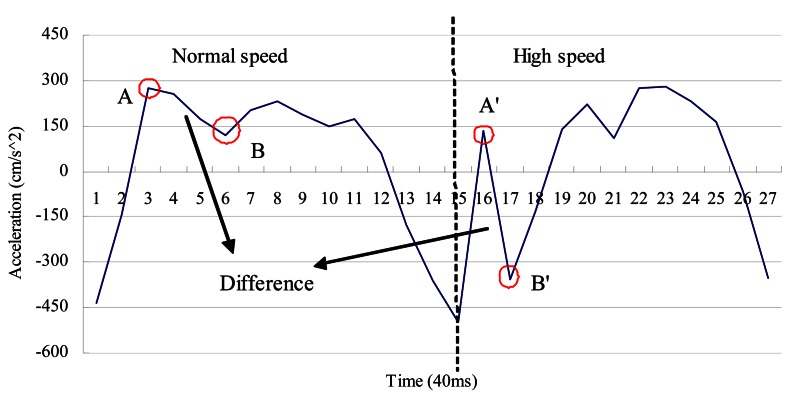
The difference between normal and high speeds.

**Figure 12.**
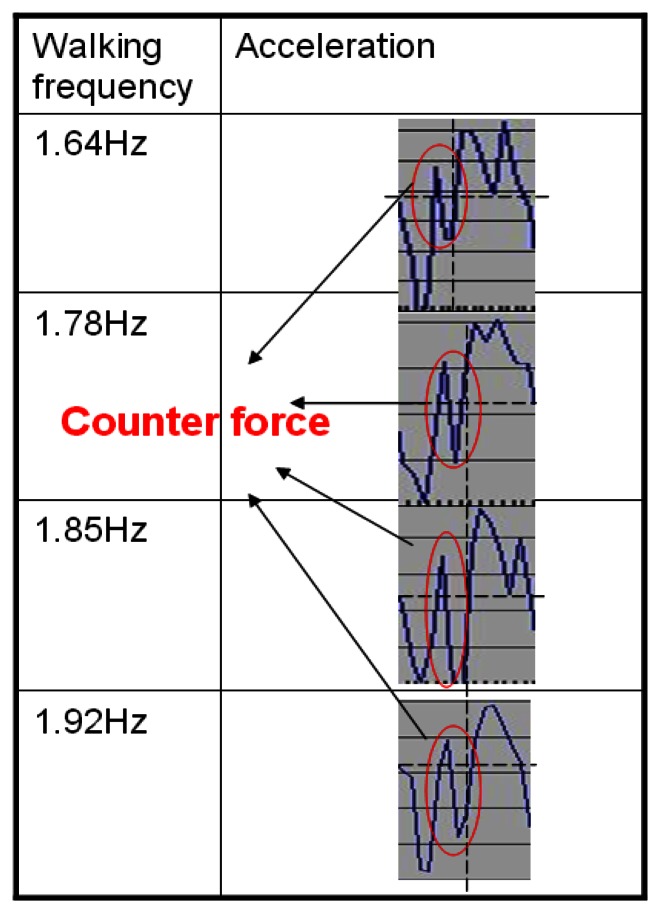
The acceleration at different high speeds.

As shown in Figure 9(b), when one walks at a normal speed, the stance event generally occurs as the first peak after the heel-touching-event. However, during high speed movement, we observe that the counter-force from the ground could introduce *an extra pulse* between the heel-touching-ground and stance events, as shown in Figure 11 (points A′ and B′) and Figure 12. Without taking this into consideration, our system could reset the velocity at the wrong point. Previous studies in kinesiology [37,62] showed that the duration from the heel-touching-ground event to the stance event normally accounts for at least 16.7% of the time in a step. Based on this observation, we can identify the right point where the stance event occurs and reset the velocity when calculating the step length.

When one is moving on the same spot, the acceleration data should not be considered in the calculation of step length, and the height of the waist does not usually change significantly. Therefore, we use a simple threshold-based filter to detect this phenomenon by looking at whether the acceleration data in all three directions (vertical, horizontal, and lateral) are lower than a certain threshold ε, as shown below, and then reset the vertical acceleration accordingly (in our implementation, *ε* is set to 1 m/s^2^):

(8) verticalAcc=0,if|lateralAcc|<ɛ&|horizontalAcc|<ɛ&|verticalAcc|<ɛ

## 4. Gyroscope Error Calibration with Map Matching

**Figure 13.**
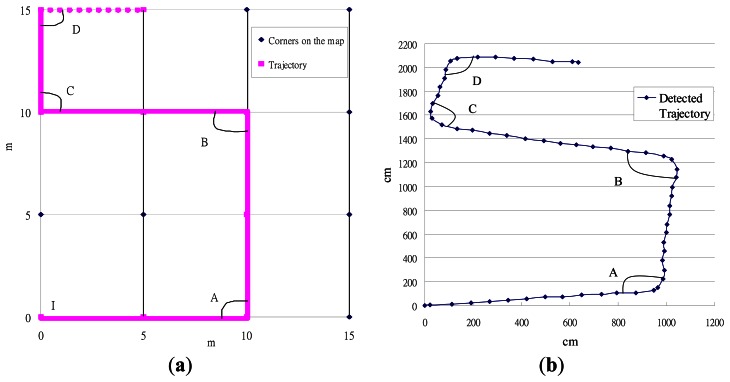
(**a**) Link-model of the map and the ground truth (**b**) the estimated trajectory from the sensor data.

**Figure 14.**
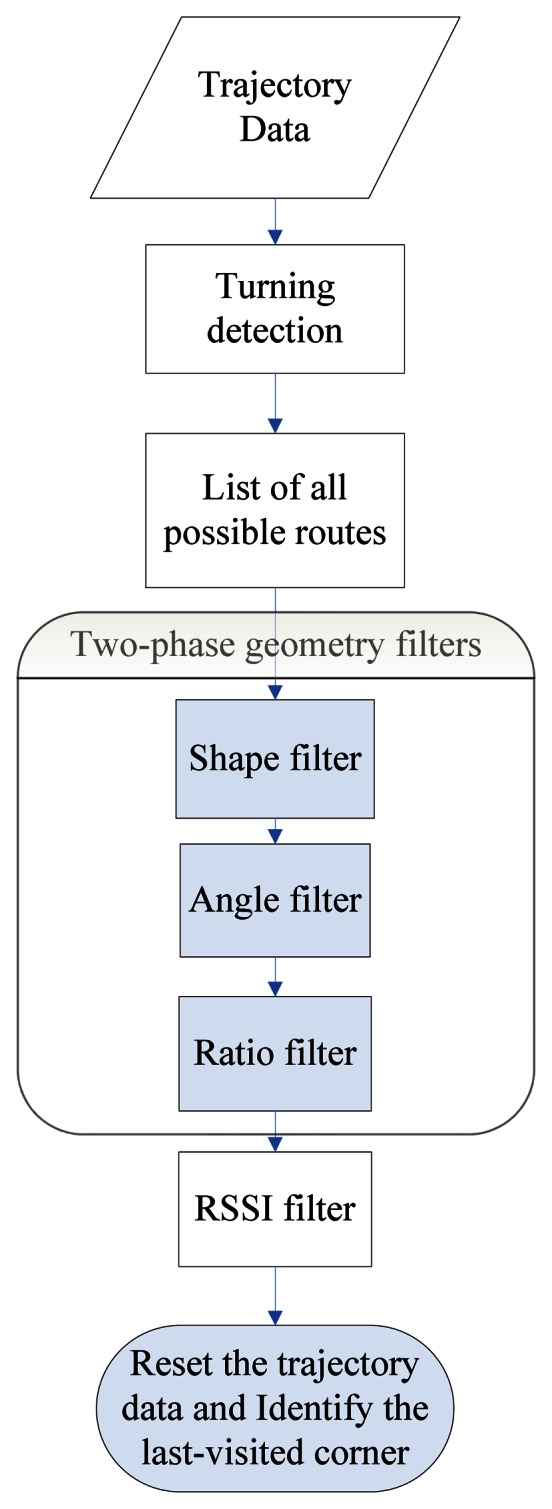
The flow chart of our map-matching algorithm.

The effectiveness of a PDR system lies in its success in accurately estimating the user's moving distance and direction. In the previous section, we discussed how to use zero velocity updating to reduce the sensor drift error when measuring the distance. In a PDR system, the direction in which the user is heading is most commonly obtained from a gyroscope sensor. However, a gyroscope can only produce the relative angular displacement (RAD) of a device with respect to a specific direction, and this is not necessarily the absolute direction. Therefore, while we could track the user's trajectory using the gyroscope, this trajectory might be biased by the error in its initial direction and appear as a rotated version of the true path, as in the example shown in Figure 13. When the error of the gyroscope is significant, and if left uncorrected, it can make the entire PDR system unusable. Map-matching is the process of comparing the pedestrian's trajectory data with a digital map of the environment to match the trajectory data to the route segment on which the pedestrian is walking, and it can be used to correct the heading error of a PDR system [63]. Unlike previous map-matching approaches that require the use of a detailed scaled map (*i.e.*, with detailed distance information for each corridor on the map) [35,36], which is normally difficult to obtain in practice, in this study we propose a new map-matching method utilizing the more commonly-seen building floor plans to calibrate the gyroscope errors. We assume that a floor plan is an “approximate” scaled down version of the physical layout of the floor. Our basic idea is to utilize the geometric similarity between the trajectory data and the floor plan to infer the last-visited corner by the user. The flow chart of our algorithm is shown in Figure 14.

**Figure 15.**
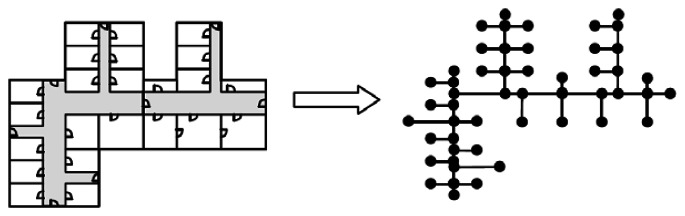
The link-node model from a floor plan.

Before starting the map matching, we adopt an approach similar to that in a prior work [64] by first converting the floor plan into a link-node model in which information such as the turning angles of the corners and comparative ratios of the lengths between any two corridors can be estimated, as shown in Figure 15. The link-node model is used to approximate the layout of corridors and corners. We then compare the geometry of the user trajectory with the link-node model to find the possible routes that the user has travelled. Here we consider the map and the trajectory as two independent graphs, say *M* and *T*. We list out all the sub-graphs of *M* and compare *T* with all these sub-graphs to find the most similar one. We define the “similarity” between two graphs by comparing their shapes, vertex angles and relative edge lengths. Once a unique route is identified, this can then be used to calibrate the trajectory data and identify the most recently visited corner. Since the location of every corner is known within the floor plan, the system can then locate the user while they move between corners using the dead-reckoning data from the accelerometer, as previously discussed in Section 3. Note that, given that the link-node model is only an approximation of the physical layout of the building (e.g., the link length might not be an exact scaled down version of the corridor length), the results of this comparison between the map and trajectory could generate multiple candidate routes. Therefore, we also implement an RSSI-based filter by using the existing WiFi-signal-based landmarks (e.g., a corridor-corner may overhear a unique set of WiFi APs, but the set may change at short distances away from that spot; some dead spots inside a building may not overhear any WiFi signals, which by itself is a signature). When a WiFi AP signal is available, we can use this RSSI-filter to select the correct route from multiple candidates. For example, if we know the user has passed a certain landmark, we can remove those candidate routes that do not contain it. This approach is similar to the method used by another recent study [65]. The map-matching process is performed every time the system detects that the user makes a turn. Details of the turn detection process are described below.

### 4.1. Turn Detection

**Figure 16.**
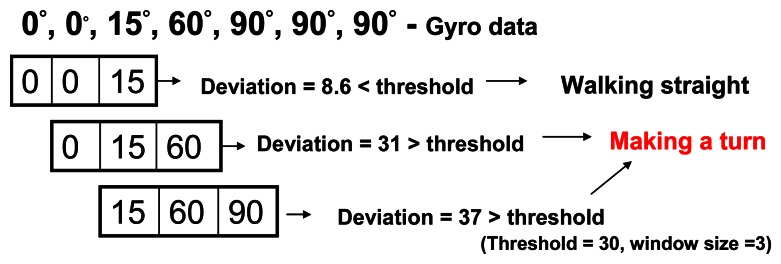
An example of turning detection.

The flow chart of our map matching algorithm is shown in Figure 14. The first step is to find out all the turnings in the trajectory data and use these as a signature to match all possible routes on the map. However, given that the gyroscope can only provide the relative angular displacement (RAD), we use a sliding-window-based algorithm to infer the user's possible turnings from the data. To determine if a person is making a turn, we compare the standard deviation of the window with a threshold which is estimated during the period when the user is walking straight. A turning event is considered to have occurred when the standard deviation of the window exceeds the threshold. Note that, since it could take several steps to turn around a corridor corner, we record all the turning events that are related to a possible corner-turning event in order to compute the angle of making a complete turn of this corner. One example is shown in Figure 16. The turning angle (*CT*) of a possible corner can be estimated as:

(9) CT=AT−BT

Here *AT* is the heading angle after making a turn around a corner and *BT* is the heading angle before making a turn around a corner. For the example in Figure 16, *CT* = *AT* – *BT* = 90 – 0 = 90. However, in reality, it is not necessary that one only makes a turn when encountering a corner. For example, one might walk back and forth along the same corridor/aisle. In addition, possible gyroscope drift errors can also produce false turning events. Therefore, detection of a turning event is not necessarily an indication that the user is indeed passing a corner. We consider these kinds of turning events, which are detected when the user is not passing a corner, as ‘fake’ turnings. Nevertheless, it is difficult to distinguish normal corner turnings from fake one based only on accelerometer and gyroscope data. In this study we utilize the floor map information to resolve the issue of fake turnings, as follows. We assume that, once the fake turnings are removed from the trajectory data, the trajectory data should be geometrically similar to a possible route on the map.

### 4.2. Filter Mechanism

We first try to find all the possible routes that a user might take (say, R_T_) based on all the possible combinations of detected turnings from the trajectory data. We then compare these R_T_ with the all the possible routes on the map (say, R_M_). The objective here is to find an (R_T_, R_M_) pair which is “*the most geometrically similar*”. We use some methods from image processing theory to solve this problem. We first model the map as a graph, *G_M_(V_M_,E_M_). V_M_* is the corner of map, such as A in Figure 13(a), and the *E_M_* denotes the corridor between two adjacent corners. We also define the graph *G_Mi_(V_Mi_, E_Mi_)* as the sub-graph of *G_M_*, which is used to model all possible routes on a map. In addition, we model the user trajectory as a graph *G_T_(V_T_, E_T_)*, where *V_T_* stands for an ordered set of detected turnings (including fake ones), such as A′,B′,C′,D′ in Figure 13(b), and *E_T_* is the edge set whose element is the connection between two adjacent detected turnings. That is, assuming *V_T_* = {*V_1_*, *V_2_*, …, *V_k_*}, *V_k_* is the *k-*th detected turning, then $E_{T}=\left\{\bar{V_{1}V_{2}},\bar{V_{2}V_{3}},...,\bar{V_{k-1}V_{k}}\right\}$. We next define the graph *G_Tj_*(*V_Tj_, E_Tj_*) as follows. There exists a set *V′* which is the power set of *V_T_* (the set that contains all subsets of *V_T_*), *i.e.*, *V′*= {ϕ, {*V_1_*}, {*V_2_*},… {*V_1_*, *V_2_*}, {*V_1_*, *V_3_*},… {*V_1_*, *V_2_*, *V_3_*,…*V_k_*}}. Here we let *V_Tj_* be an ordered set, *V_Tj_* ∈ *V′* and *|V_Tj_*| > 1. The *E_Tj_* is the edge set which contains the edge between any two adjacent elements in *V_Tj_*. In other words, *G_Tj_* is used to model all possible routes that could be generated based on the detected turnings (including fake ones), and *G_Mi_* stands for the accessible route on the map.

Again, the idea here is to find a (*G_Mi_*, *G_Tj_*) pair which is the most geometrically similar. In other words, we want to eliminate those hypothetical routes generated in *G_Tj_* that cannot be found on the real map. We consider two graphs are geometrically similar if they have similar shapes, angles (*i.e.*, the angle between two connected edges) and edge lengths (after normalization). We design a two-phase filtering mechanism and employ three filters: shape filter, angle filter and edge filter. In phase one, we input all (*G_Mi_*, *G_T_*) pairs into these three filters to remove those which are not geometrically similar. The purpose of phase two is to remove the non-existing routes caused by fake turnings, and we input all (*G_Mi_*, *G_Tj_*) pairs into these filters to remove the non-existing routes, as shown in Figure 14.

The aim of the above two-phase filtering mechanism is to produce a (*G_Mi_*, *G_Tj_*) pair which is geometrically similar. However, when we do not have “*sufficient*” trajectory data (e.g., when there is no detected turning in the trajectory data), it is possible that we can still have multiple candidate routes remaining after the geometry-similarity filtering. Therefore, when multiple candidate routes exist, we employ an RSSI-based filter by using the existing WiFi-signal-based landmarks, as described previously, to further select the correct one among multiple candidates. Next, we discuss the details of each filter.

#### 4.2.1. The Shape Filter

**Figure 17.**
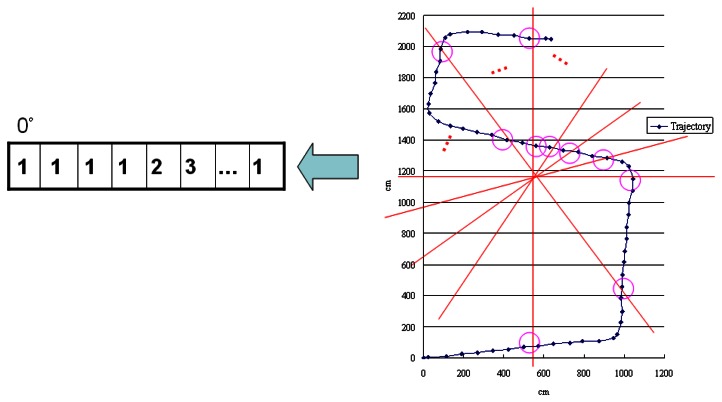
Example of the shape filter.

We adopt the idea of a shape descriptor [66,67] to implement our shape filter by comparing the shapes of two graphs. Considering the nature of our input data and the computational overhead, we modify the original shape descriptor method to suit our scenario. To reduce the computational overhead, we calculate the centroid [68,69] of each graph and create a line every 10 degrees from 0° to 180° to pass through this. We then calculate how many crossing points on the edge can be made by each line and record them in a one-dimensional array, as shown in Figure 17. Finally, we compare the arrays generated based the two graphs to determine their similarity using Euclidean distance (*L-2* norm) [70]. We set a threshold to judge the similarity between these two graphs. If the value is over the threshold, the system will consider these two graphs are different and remove them from the set of candidates.

#### 4.2.2. The Angle Filter

**Figure 18.**
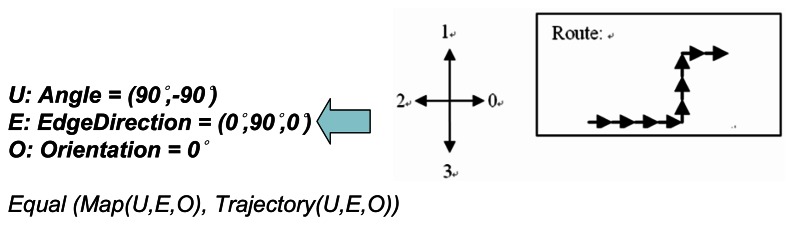
Features of the angle filter.

We adopt the concept of chain code [71] to implement the angle filter. The chain code uses a sequence of numbers to represent a series of different moving directions and transform a graph to a one-dimensional expression. However, we cannot directly apply a full-fledged chain code to our system. First, it is difficult to decide the number of sampling points for the trajectory data, since each step distance could be different. Second, the computational overhead of using the chain code is proportional to the number of steps in the trajectory data and the number of candidate routes on the map. Given that what we consider here is a real-time localization system and the computation capability of a smart-phone is limited, we cannot just naively use the chain code. Therefore, we adopt the concept of the chain code and implement it separately via two different filters: the angle filter and the edge filter. Conceptually, the outputs of the chain code include the angle information (e.g., *A* and *A′* in Figure 13), the direction of the edge and the normalized edge ratio (*i.e.*, we divide the distance of each edge by the distance of the longest edge). In our angle filter, for example, we compare every matching angle and the direction of each edge between *G_T_* and *G_Mi_*, and use a threshold to determine whether they are all close enough, as shown in Figure 18. After filtering out those *G_Mi_* which do not have similar angles, we then implement the 2nd part of the chain code with an edge filter, as described below.

#### 4.2.3. The Edge Filter

With this filter, we check whether the normalized edge ratios between two graphs, for example, *G_Tj_* and *G_Mi_*, are similar or not. The system calculates the displacement between any two adjacent vertices in the graph and stores these displacements as a vector.

**Figure 19.**
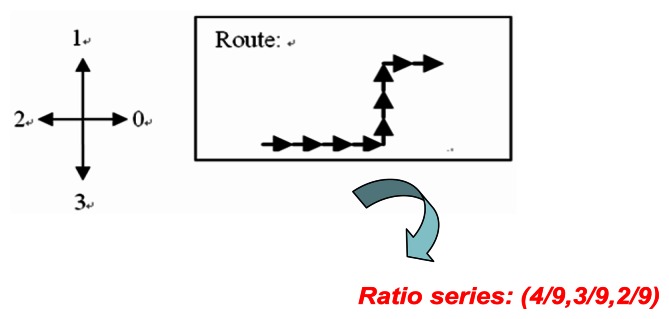
The input to the edge filter.

Figure 19 shows an example input to the edge filter. We then use the Euclidean distance and set a threshold to determine whether the vectors produced for *G_Tj_* and *G_Mi_* are similar or not. If the value of the *L-p* norm of these two graphs is over the threshold, the system then removes the corresponding (*G_Tj_*, *G_Mi_*) pair from the candidates.

## 5. Evaluation

In this section, we discuss the performance of our PDR-based localization system. We first discuss the results of our step length estimation method, and then show the performance of the above-mentioned map matching algorithm.

### 5.1. Experiment Setup

**Figure 20.**
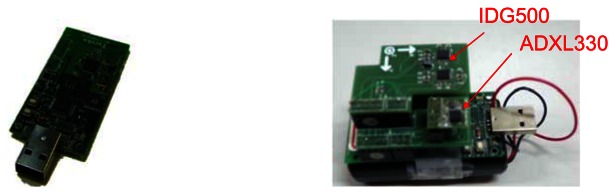
The Taroko with ADXL330 and IDG500.

We use two different sensor platforms in our experiments: Taroko motes and a variety of Android phones from HTC and Samsung. Taroko is a modified version of the TelosB mote [72], which was originally designed by UC Berkeley. Taroko is a programmable, low-power wireless sensor platform. The Taroko microcontroller unit is a TI MSP430 F1611 with 16 bit RISC [73]. MSP430 has 48 K bytes flash memory and 10 K bytes RAM that supports serial communication formats such as UART, I2C, SPI, and Digital I/O. Taroko is also equipped with a CC2420 RF transceiver [74], which is a low cost device for wireless communication in 2.4 GHz based on IEEE 802.15.4 [75]. The maximum radio distance is around 100 m. Taroko also supports the USB interface using an FTDI chip [76], and can use this to connect to a computer for powering, program upload and data collection. Taroko can accommodate different sensor components, e.g., temperature, humidity, infrared, accelerometer, gyroscope, and magnetic. In this work, we use an extended board to combine the accelerometer (ADXL330 [77]) and gyroscope (IDG500 [78]), as shown in Figure 20. The ADXL330 is a small, low power, 3-axis accelerometer with signal conditioned voltage outputs, all on a single monolithic IC. It can measure acceleration with a minimum full-scale range of ±3 g. The IDG-500 is an angular rate sensor and its full scale range is ±500°/s. It uses the MEMS technology with vertically driven, vibrating masses to make a functionally complete, low-cost angular rate sensor. All the required electronics are integrated onto a single chip with the sensor.

We first implement our algorithms on Taroko motes for ease of debugging. We then port the same algorithms into smart-phones after testing them on the motes. We perform two set of experiments on the smart-phones. One is placing the smart-phone in a shirt pocket and the other is putting it in a pants pocket. We use a laser distance meter to measure the actual travel distance of the user. In addition, to record the user's actual location, we pasted markers on the ground at precisely known locations. Each of these markers had a number on it, and the user recorded the numbers when they walked passed them. The results based on the Taroko motes are similar to those from the smart-phones.

### 5.2. Step Length Estimation

**Figure 21.**
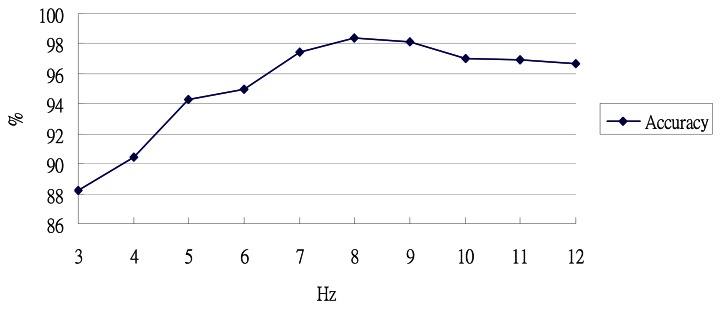
The accuracy using different cut-off frequencies for the low pass filter.

As discussed previously, we use a low-pass filter to filter out the noise in our step length estimation algorithm. We choose 8 Hz as our cut-off frequency for filtering the noise. To examine whether this chosen frequency produces the best results, we perform a set of experiments using different frequencies for the low-pass filter, ranging from 3 to 12 Hz, and compare their accuracies in estimating step length. As shown in Figure 21, the use of 8 Hz as the cut-off frequency produces the most accurate results.

Some of the state-of-the-art pedometers on the market can also output walking distance. We compare our method with these pedometers and find that they only achieve up to 90% accuracy, which is significantly lower than our results (98.25%). Furthermore, as discussed previously, our approach is based on the idea of using the change in height of the waist to estimate step length, which is similar to the method used in Weinberg [31]. He proposed an equation to estimate distance by observing the vertical acceleration during walking. We implement Weinberg's method and find that its accuracy is about 96.7%. However, one limitation of Weinberg's approach is that its system parameters need to be re-trained for every new user.

In our system, we use a low-pass filter (LPF) to filter out the noise and employ zero velocity update (ZUPT) to calibrate the drift error of the accelerometer. To understand the effect of these mechanisms, we enable only one of them at a time. As shown in Table 1, ZUPT has a higher impact on the system performance than the implementation of the low-pass filter. When ZUPT is disabled, the accuracy drops from 98.25% to 84.1%.

We also compare our results with those from existing foot-mounted PDR systems. As shown in Table 2, the performance of our method is close to that of the state-of-the-art foot-mounted approach [17]. Note that, although the foot-mounted method can be used to estimate step length, it is generally not good at estimating the direction a person is heading in, as noted previously.

**Figure 22.**
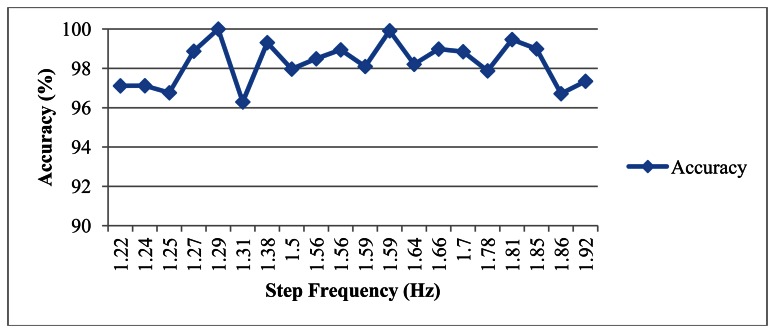
Accuracy of estimated distance under different walking speeds.

Considering the variations that might exist in different individuals' walking gaits, we performed an experiment in which we asked the subject to walk with different speeds, as shown in Figure 22. Here the step frequency is defined as the number of walking steps in one second. The average accuracy is about 98.26% and standard deviation is about 1.09%. In addition, we asked two other persons (one male and one female) to perform the same experiment for the walking distance measurements. Each experiment, again, is repeated for 10 times. Their average accuracy and standard deviation are (98.42%, 0.98%) and (97.93%, 1.86%) respectively, which are similar to the results shown in Table 1.

### 5.3. Map Matching

**Figure 23.**
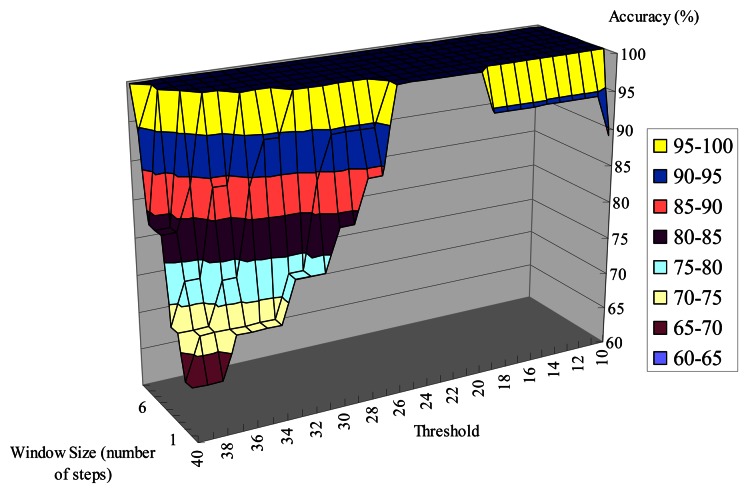
The number of walking steps between two corners is more than the window size.

**Figure 24.**
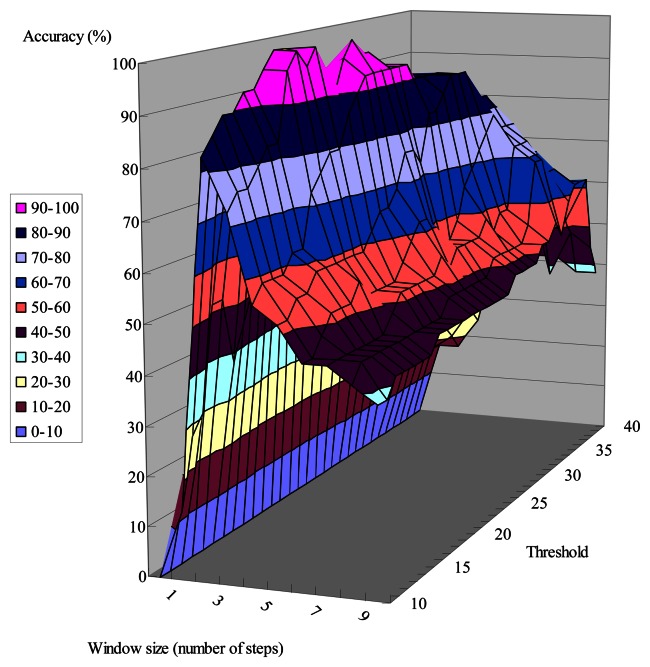
The number of walking steps between two corners is less than the window size.

As discussed above, we use a sliding-window-based algorithm to detect the user's possible turnings from the gyroscope data. To determine if a person is making a turn, we compare the standard deviation of the window with a threshold. Generally, when the chosen window size is too small, all the walking steps that happen during a turning might not be able to be included within a window. On the other hand, when the window size is too big, two different turnings can be included in the same window if the number of walking steps between two corners is less than the window size. As shown in Figures 23 and 24, the accuracy of detecting turnings becomes lower when the chosen window size is too small or too big. Therefore, in our experiments we choose the window size from the range defined below, in which *D* is the shortest distance between two adjacent corridors:

(10) $$2<\text{WindowSize}<\frac{D}{\text{AvgStepLength}}$$

The threshold is obtained using the standard deviation of the window during the period when the user is walking straight. In the shape filter, we set a line, every 10 degrees from 0° to 180°, that passes through the centroid of the graph, and use the crossing points on the edges obtained in this way to determine if two graphs have similar shapes. To understand the effects of the gaps between two crossing lines (default 10°) on determining shape similarity, we vary this gap from 0° to 70°. Generally, the system will have less computational overhead when the gap is larger. However, a larger gap also suggests that poorer results will be obtained, since fewer crossing points will be generated for the comparison of shape similarity.

**Figure 25.**
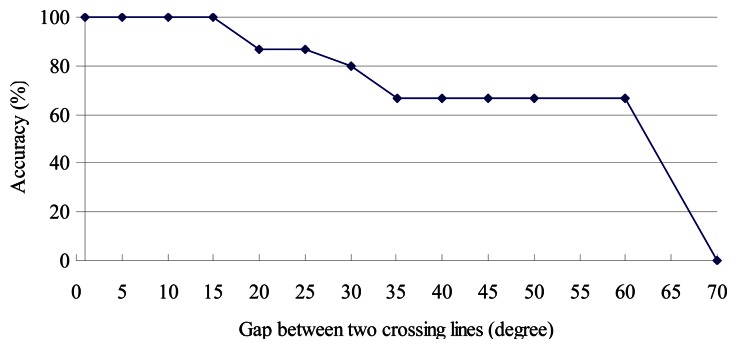
The discrimination of different slopes.

As shown in Figure 25, we start getting inaccurate results when the gap is larger than 15°.

**Figure 26.**
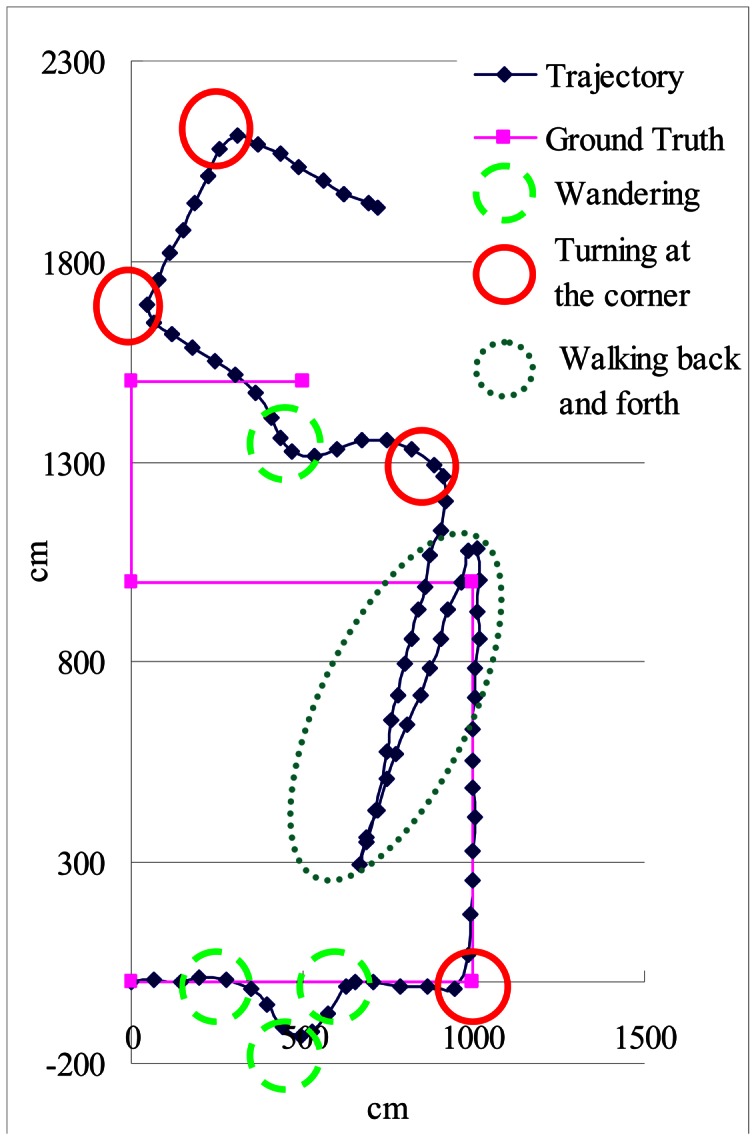
The testing environment and route.

Finally, to test the performance of our localization system, we choose a route which is about 40 meters long and includes four corridors and corners, as shown in Figure 26. We deliberately created some ‘fake turnings’ by having the user wander about around at certain spots, shown as the dashed circle in Figure 26. The solid circles in Figure 26 indicate when the user made a turn at the corner. We repeated this experiment 10 times and our results show that our location error is about 0.48 meter, and the standard deviation is about 0.43 meter.

## 6. Conclusions

In this paper we propose a novel method to accurately estimate the step length of the user based on the change in waist height. We use an accelerometer to measure the user's instant change in height, and utilize the characteristics of simple harmonic motion during walking to calibrate the drift errors of the accelerometer when calculating the change in height. Based on the Pythagorean Theorem, we can then estimate each step length using the user's change in height. Furthermore, we design a map matching algorithm to calibrate the direction errors from the gyroscope using building floor plans, which are readily available. Our map matching algorithm implements three filters, namely the shape filter, angle filter and edge filter, to infer the user's last-visited corner. Our results show that we can achieve about 98.26% accuracy in estimating the user's walking distance, while the overall location error in our experiment is about 0.48 meters.

## Figures and Tables

**Table 1.** Comparison with other waist-mounted methods and the influence of the mechanism.

	**Pedometer**	**Weinberg**	**Ours (A:LPF, B:ZUPT)**			
**Method**	Constant step length × step number	$k\times n\times \sqrt[{4}]{Acc_{\max}-Acc_{\min}}$	Original (A+B)	A	B	None
**Accuracy (Estimation/Ground Truth)**	90.09%	96.78%	98.25%	84.1%	95.29%	75.53%
**Standard deviation**	1.83%	3.18%	1.29%	7.41%	2.22%	16.3%

**Table 2.** Comparison with foot-mounted PDR.

	**Our Approach**	**Foot-mounted method (L. Ojeda 2007)**
**Hardware**	3-axis accelerometer & gyroscope	6-DoF inertial sensor (accelerometer + Gyro + Magnetic)
**Sample Rate**	25Hz	200Hz
**Placement**	Waist	Foot
**ZUPT**	Use the concept of SHM	Calculate the angular velocity to detect zero velocity
**Accuracy**	98.25±1.29% (total distance)	>98% (total distance) >99% (coordinate)
